# Application of Fourier Transform Infrared (FTIR) Spectroscopy in Characterization of Green Synthesized Nanoparticles

**DOI:** 10.3390/molecules30030684

**Published:** 2025-02-04

**Authors:** Sylwia Pasieczna-Patkowska, Marcin Cichy, Jolanta Flieger

**Affiliations:** 1 Department of Chemical Technology, Faculty of Chemistry, Institute of Chemical Sciences, Maria Curie-Skłodowska University, 3 Maria Curie-Skłodowska Sq., 20-031 Lublin, Poland; marcin.cichy@mail.umcs.pl; 2 Department of Analytical Chemistry, Medical University of Lublin, Chodźki 4A, 20-093 Lublin, Poland; j.flieger@umlub.pl

**Keywords:** FTIR, nanoparticles, nanoparticles characterization, green synthesis

## Abstract

The fundamental principle of Fourier Transform Infrared (FTIR) spectroscopy is based on the vibration and rotation of atoms, and it has become a universal and widely used spectral methodology for the detection of internal molecular structures in a diverse range of fields. A considerable number of review articles pertaining to the applications of FTIR spectroscopy have been published in recent years. Nevertheless, a comprehensive summary of the application of FTIR spectroscopy in nanoparticles’ (NPs’) green synthesis has yet to be presented. In the present paper, we propose a series of case studies that demonstrate the application of FTIR spectroscopy in the analysis of metal and metal oxide NPs that have been synthesized using green synthesis processes. Furthermore, a summary is presented of the position of functional group bands in FTIR spectra that are responsible for the reduction, capping and stabilization of NPs. In this review, we explore the advantages and limitations of FTIR and propose methodologies for overcoming these challenges. We also present potential solutions for the analysis of complex FTIR spectra. The present summary is intended to serve as a compendium of information for researchers engaged in the field of green synthesis of NPs, utilizing FTIR spectroscopy as a research tool.

## 1. Introduction

Nanotechnology represents one of the most significant and active areas of investigation within the broader field of modern materials science. Nanoparticles exhibit novel or modified properties that are attributable to specific characteristics, including size, morphology and distribution. In recent years, there have been significant advances in the synthesis of nanoparticles (NPs) of defined size and morphology for specific applications [1]. The extensive practical utilization of nanoparticles (particles of small size falling in the nanoscale range of 1–100 nm) is attributed to their numerous rare, unique and fascinating characteristics, which are valued more than their bulk counterparts [1,2]. The primary objective is to refine the methodologies employed in the synthesis of NPs with a specific size, shape, composition and ordered dispersion, which influence their physical, chemical, catalytic, optical, magnetic, electronic and electrical properties [3,4,5]. This renders them ideal candidates for environmental, biomedical and biotechnological applications.

In order to meet the growing demand for environmentally friendly nanoparticles, researchers have employed diverse strategies to synthesize a variety of metal and metal oxide nanoparticles. Physical and chemical techniques are typically employed in the synthesis of nanoparticles, although the former are often prohibitively expensive. However, the latter are known to have adverse effects on the environment and living organisms. The development of these synthesis techniques for large–scale production is constrained by the high costs associated with high energy consumption; the use of hazardous chemicals and harmful organic solvents; the generation of unreliable intermediates; and the production of harmful, toxic by–products, which contribute to environmental pollution and numerous biohazards. Conversely, green synthesis methods present a viable solution, employing bio–based materials such as plants, microorganisms (bacteria, algae, fungi, yeast) and their enzymes and extracts, isolated sugars and agricultural waste as environmentally friendly sources for nanoparticle synthesis [6,7,8,9]. The concept of green synthesis is predicated on the following principles: the implementation of a reliable and eco–friendly pathway with mild reaction conditions, the utilization of non–toxic precursors and the production of a lower amount of waste, with the objective of ensuring a sustainable environment. Consequently, the design and synthesis of novel products that are safe, reusable and biodegradable is now possible. A substantial body of evidence demonstrates that green synthesis, usually single–step methods, effectively produce nanoparticles with desirable properties. The synthesis of well–defined metal and metal oxide nanoparticles requires precise control of several key variables, including concentration of extract/biomass and metal salt, pH of the reaction mixture, incubation time and temperature. The synthesized nanoparticles can be characterized by a number of analytical techniques, including UV–visible spectrophotometry (UV–Vis), transmission electron microscopy (TEM), scanning electron microscopy (SEM), energy–dispersive X–ray spectroscopy (EDX), X–ray diffraction (XRD), X–ray photoelectron spectroscopy (XPS), dynamic light scattering (DLS), Raman spectroscopy and Fourier–transform infrared spectroscopy (FTIR). UV–Vis provides information about nanoparticles based on their optical properties. The magnitude, peak wavelength and spectral bandwidth associated with NPs are contingent on the particle’s size, shape and material composition [10]. However, it does not offer insights into the chemical structure or functional groups present in the NPs. TEM is utilized to obtain high–resolution images of NPs, allowing for the determination of their size, shape and distribution. SEM complements TEM by providing detailed surface morphology images of nanoparticles. EDX is often coupled with SEM to provide elemental analysis of NPs. XRD is employed to determine the crystalline structure and phase purity of NPs. XPS survey spectra give information regarding qualitative and quantitative surface composition, typically of 3–5 nm depth, while high–resolution spectra offer insights into functional groups present on NPs surfaces. DLS measures the size distribution and stability of colloidal suspensions by analyzing fluctuations in scattered light intensity due to Brownian motion. This technique is particularly useful for determining the hydrodynamic diameter of nanoparticles in solution, providing insights into their stability over time. In some cases, all previously mentioned techniques are exclusively employed for the study of a particular property, whereas in others, they are utilized in combination. A comprehensive characterization of the NPs under investigation can only be accomplished by combining the information obtained from these various techniques [11,12]. This multifaceted approach has the potential to elucidate the relationship between the chemical composition of bio–based extracts and the resulting NPs characteristics.

Raman and FTIR spectroscopy are techniques employed for the identification of molecular structures and the characterization of chemical bonding in synthesized nanoparticles. Both techniques provide insights into the vibrational modes of molecules, which can indicate the presence of specific functional groups and the chemical environment surrounding NPs. Raman spectroscopy detects subtle molecular vibrations of molecules with polarizability, while FTIR spectroscopy provides detailed information about molecular bonds and functional groups that have a permanent dipole moment. However, Raman spectroscopy can sometimes induce fluorescence or damage samples due to high–energy laser excitation, particularly with sensitive biological samples. In comparison, FTIR employs longer wavelengths, which are generally less harmful to the samples. This characteristic enables the execution of repeated analyses without compromising the integrity of the samples. The intensity of Raman peaks is typically weaker than that of FTIR because it relies on changes in polarizability rather than direct absorption. This results in generally lower signal strength, requiring longer acquisition times or higher sample concentrations to achieve comparable peak intensities. FTIR spectroscopy proved advantageous in the analysis of bioorganic surface–associated components, whereas Raman spectroscopy exhibited heightened sensitivity to variations in the allotropic modifications and crystalline structure of NPs [13].

FTIR spectroscopy is exceptionally versatile, with applications spanning multiple domains, including materials science, biology and environmental studies. Its ability to analyze complex mixtures without extensive sample preparation makes it particularly valuable for studying nanoparticles and their interactions with biological systems. Key applications include identification of functional groups and molecular structures in nanoparticles, aiding in the understanding of their chemical properties and potential applications in fields like drug delivery (including characterization of drug–polymer interactions [14,15], monitoring of drug loading and release, interaction of nanoparticles with biological tissues and cells [14,16,17] and quality control and process validation [18]), environmental remediation and degradation of pollutants [9,19,20,21,22] and interactions between nanoparticles and biological entities, such as bacteria [23,24]. FTIR spectroscopy has emerged as a pivotal technique in the field of nanoparticle science, providing detailed insights into the molecular composition and interactions of materials at the nanoscale. The employment of FTIR in the analysis of green synthesized NPs is driven by numerous crucial factors associated with their synthesis, stabilization and application. FTIR analysis has the capability to discern specific absorption peaks corresponding to various functional groups, thereby signifying their involvement in the reduction of metal ions, or the formation of metal oxide NPs and their subsequent stabilization. The presence of characteristic peaks in the FTIR spectrum serves to substantiate the efficacious synthesis of nanoparticles by indicating the existence of biomolecules that contribute to nanoparticle formation. Furthermore, the interactions between the synthesized nanoparticles and the capping agents can be studied through FTIR, which provides insights into how these interactions influence particle stability. This understanding is essential for ensuring that the NPs maintain their desired properties over time, making them suitable for various applications such as drug delivery and bioremediation.

The objective of this review is to collate and organize the latest information on the application of FTIR spectroscopy in the analysis of nanoparticles produced via green synthesis, as well as to establish a systematic framework for the spectral information on the functional groups that may be responsible for the reduction, capping and stabilization of nanoparticles. This review explores the diverse applications of FTIR spectroscopy in the green synthesis of metal and metal oxide nanoparticles, highlighting difficulties encountered by researchers when analyzing the FTIR spectra of NPs.

## 2. FTIR Spectroscopy—Fundamentals, Techniques and Limitations

Fourier–transform infrared spectroscopy operates on the principle that molecules absorb infrared light at specific frequencies, which correspond to the vibrational modes of their chemical bonds. The resulting absorption spectrum serves as a molecular fingerprint, enabling identification and characterization of various organic and inorganic substances. The parts of molecules that determine the main chemical properties of compounds, i.e., functional groups, absorb IR radiation in the same wavenumbers range even though they are part of different compounds [25]. This means that there is a clear relationship between the wavenumber at which a molecule absorbs IR radiation and the structure of the material being tested, so it is possible to identify the substance by recognizing the bands in the IR spectrum that correspond to the functional groups in tested material. This makes infrared spectroscopy a useful tool in chemical analysis. The most commonly used spectral region for analytical purposes is the mid–infrared (mid–IR), covering the range 4000–400 cm^−1^. A detailed description of the theory and working principles of FTIR is provided by Smith [25].

FTIR spectroscopy relies on the interaction of infrared radiation with molecular vibrations that create a dipole moment. Pure metals, being composed of metallic bonds, do not have the necessary molecular structure to produce significant dipole moments. As a result, they do not absorb infrared radiation effectively, leading to a lack of informative spectral data. However, FTIR spectroscopy can be used for the characterization of molecular adsorbates on metal particles.

Improvements in FTIR instrumentation have led to the development of a wide range of new sensitive techniques to examine samples that were previously difficult to analyze. Among various FTIR spectroscopy techniques transmission spectroscopy (TS), attenuated total reflectance (ATR) and photoacoustic spectroscopy (PAS) are the most commonly used. Each technique is designed to study different types of materials and has its own advantages and disadvantages, while the quality of the spectrum depends, among other things, on the appropriate choice of technique, light source, detector, etc. It is therefore extremely important to determine the suitability and adapt the method of analysis to the specific case. The development of diverse FTIR techniques has significantly diminished the time required for sample preparation, rendering them a compelling option for laboratories and other practical applications.

### 2.1. Transmission Technique (TS)

The transmission technique (TS) is one of the most commonly employed IR spectroscopy techniques. The TS technique offers high–quality spectra with good signal–to–noise (S/N) ratio and good reproducibility, and is considered satisfactory for qualitative and semi–quantitative analysis. In this regard, it should be noted that the TS technique fails to measure the precise quantity of a substance; rather, it expresses the results as an estimate of the presence of a detected substance.

The main drawbacks of the results obtained by using the transmission technique primarily lie in the sample preparation procedure. Thus, one of primary TS limitations is the necessity to prepare pellets from a mixture of the studied material and compound transparent to infrared radiation (simultaneously serving as a matrix; usually potassium bromide, KBr) within the spectral range employed. The analysis of opaque, plant–derived or carbonaceous materials using FTIR transmission spectroscopy is challenging due to the fact that such materials, being optically opaque, may absorb incident IR radiation to a significant extent. The preparation of samples for analysis by grinding and diluting with KBr can result in the destruction of their structure. Furthermore, potassium bromide is a hygroscopic compound, which means that the prepared pellets may contain some moisture. Water is visible in the IR spectrum as a broad band with maxima at ~3500 and ~1630 cm^−1^, and these bands are sometimes intense enough to overlap with the bands originating from the sample. This is particularly important when hydroxyl groups are the subject of study. Preparation of the KBr pellets for TS measurements is laborious, time consuming and suffers from risk of samples contamination. Therefore, in some cases, it may be preferable to select an alternative available technique.

### 2.2. Attenuated Total Reflectance (ATR)

**Figure 1:**
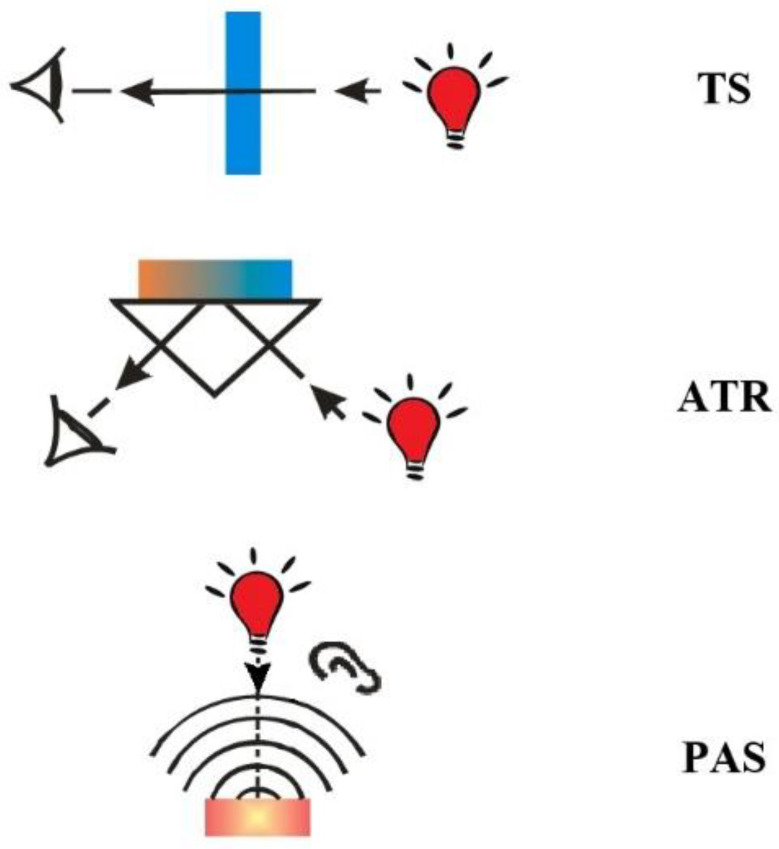
Schematic representation of FTIR techniques used for nanoparticles analysis: TS, ATR and PAS.

The ATR technique, also called internal reflection spectroscopy (IRS), enables the examination of solid samples (i.e., powders, soft solids) with smooth, flat surfaces and liquids. It is a versatile, non–destructive, high–sensitivity and broad–applicability technique that allows for the examination of samples that strongly absorb IR radiation and which would not be analysable using the TS technique. ATR measures the absorption of infrared light by a sample placed in close contact with a high refractive index (HRI) crystal (i.e., diamond, germanium, silicon or zinc selenide). When a beam of IR radiation traverses HRI material, the light is repeatedly internally reflected, resulting in the generation of an evanescent wave. Upon contact between the sample and the evanescent wave at the sample/crystal interface, the wave is absorbed by the sample, leading to a reduction in its intensity. The attenuated beam then reflects off the HRI material and is subsequently directed towards the detector. The depth of penetration of IR beam into the material under investigation is contingent upon the refractive index at the sample/crystal interface, which is in turn dependent upon the material of the crystal, the angle of incidence and the wavelength of the IR radiation [25]. ATR spectra demonstrate a relative shift in the intensity of the bands, with those at low wavenumbers exhibiting greater intensity than those at high wavenumbers. This can be corrected relatively easily mathematically (ATR correction). A more challenging issue is the shift in vibrational frequencies, which results in a shift of the peak maximum. This may result in an ambiguous interpretation of the spectrum obtained through the ATR technique. Furthermore, the manner in which the measurement is conducted can result in inadequate contact between the sample and the refractive crystal, potentially leading to inaccuracies in the obtained results [26]. It is essential to ensure that the contact between the reflecting surface and the sample is optimized to ensure the efficacy of the technique, e.g., to achieve good reproducibility of the spectra. ATR is generally much more straightforward than the TS technique, as it only requires good contact with the ATR element to produce a reliable measurement. However, there are some differences: the strength of the ATR effect is dependent on wavelength, whereas the transmission measurement is not [25], and reproducibility is better achieved in TS mode compared to ATR mode. The ATR technique is employed in two main configurations, and each of which offers distinct advantages. In Single Reflection ATR, the incident infrared light undergoes a single reflection at the crystal–sample interface (Figure 1), facilitating a straightforward and uncomplicated ATR measurement. This configuration is well–suited for routine analysis and samples with relatively high refractive indices. Conversely, in the Multiple Reflection ATR configuration, infrared light undergoes repeated internal reflections within the crystal prior to exiting, thereby increasing the effective path length and enhancing the sensitivity, particularly in cases where the sample has a low concentration or where absorptions are weak (e.g., thin–film samples).

ATR is a technique that provides information mainly from the surface of the sample being tested and limited penetration depth is also a consideration in FTIR/ATR spectroscopy. If the region of interest is located deeper within the sample, the ATR measurement may not capture the desired molecular information accurately. In such cases, alternative sampling techniques such as TS may be more suitable.

### 2.3. Photoacoustic FTIR Spectroscopy (FTIR/PAS)

Photoacoustic spectroscopy (PAS) detects light absorption (photo) by using sound (acoustic). It is founded upon the phenomenon of the photoacoustic effect, which entails the generation of an acoustic wave at the surface of the tested material as a consequence of the absorption of modulated electromagnetic radiation by this material. Sampling depth is dependent on the wavenumber of infrared radiation and decreases as wavenumber increases. In this regard, PAS is analogous to ATR, wherein the sampling depth is also contingent upon the wavenumber. In the conventional photoacoustic spectroscopy technique, the sample to be investigated is placed inside a closed chamber (the photoacoustic cell) filled with a suitable gas [27] (usually helium). While a modulated radiation source is directed towards the sample surface, the generated photoacoustic signal is detected by a sensitive microphone attached to the photoacoustic cell. Photoacoustic FTIR (FTIR/PAS) spectroscopy has been described as a non–destructive method for the sensitive analysis of solid–phase materials. As only the absorbed radiation is measured, PAS is not affected by the effects of light scattering and reflection. The principal benefit of photoacoustic spectroscopy is that it necessitates no complex sample preparation. This technique can be used in the analysis of materials that are difficult to homogenize or for which the structure or chemical composition may change during grinding. Even samples with rough surfaces and strongly scattering infrared light can be readily measured, as the photoacoustic signal is proportional to the absorbed energy, in contrast to the reflected energy observed in reflectance techniques. However, the PAS method exhibits a relatively poor S/N ratio, necessitating longer data acquisition times than those required for TS and ATR techniques [28]. It is of the utmost importance that the tested samples are kept in a dry state, as moisture could potentially cause damage to the sensitive microphone. Literature reports describing the use of PAS technique in the infrared range in nanoparticle research are still scarce [29,30,31].

PAS and to some extent ATR are not affected by the presence of water. PAS and ATR are surface techniques that allow the bulk of the sample to be distinguished from that of the sample surface [32,33]. In PAS and ATR techniques, the signal is generated at a maximum of a few micrometres from the sample surface and provides information about the chemical structure of the surface, while in the TS technique signal is derived from the entire bulk of the sample. The surface sensitivity is ~1 μm for PAS and monolayer for ATR [34]. All the FTIR techniques mentioned appear to be suitable for the analysis of nanoparticles; however, the choice is typically determined by the availability of specific accessories (ATR, PAS) rather than the suitability of the technique. Transmission spectroscopy, which does not require any additional accessories, is most frequently used to obtain IR spectra of NPs. Figure 1 depicts the schematic representation of FTIR techniques employed for the analysis of nanoparticles, namely, transmission spectroscopy (TS), attenuated total reflection (ATR) and photoacoustic spectroscopy (PAS). The advantages and limitations of each technique are summarized in Table 1.

### 2.4. Some FTIR Limitations and Potential Improvements

Notwithstanding its undisputed advantages, FTIR is not without its limitations and disadvantages in assessing green synthesized NPs. As it was mentioned, FTIR analysis is highly sensitive to the preparation of samples. Variations in sample handling, such as drying methods, the use of different matrices (like KBr) or the performance of washing steps after NPs synthesis, can lead to inconsistent results. For instance, grinding and pressing samples (using TS technique) can alter the spectral characteristics, potentially masking important information about the functional groups in tested biological samples [35,36]. As demonstrated by Kamnev et al. [35], grinding of the *Azospirillum brasilense* Sp7 and *Azospirillum baldaniorum* Sp245 biomass samples with KBr resulted in a shift towards lower wavenumbers of bands of inter– and intramolecular hydrogen bonds between the ester carbonyl group related to cellular poly–3–hydroxybutyrate (PHB) present in the samples. This finding indicated that the grinding step induced a partial transition from the metastable and more amorphous state of the intracellular PHB to its more ordered state. In order to circumvent this issue, it would be advisable to employ a methodology that does not necessitate sample preparation, such as ATR or PAS technique.

As previously mentioned, the presence of water in a sample can alter the measured FTIR spectrum. Furthermore, the H–O–H bending vibration of water (maximum at ~1630 cm^−1^) falls within the amide I region of proteins (1700–1600 cm^−1^), which underscores the necessity to dry the samples prior to FTIR analysis. Research has demonstrated [35] that the drying temperature, with a maximum limit of 45 °C in order to prevent protein denaturation, exerts a greater influence on the resulting IR spectrum than the drying duration.

In the context of the green synthesis of NPs, the washing steps have been demonstrated to be instrumental in ensuring the desired properties, stability and ultimately purity of the resulting product [36]. The process of washing serves to remove unreacted precursors, by–products and other contaminants that may interfere with the properties of the NPs. This is important for achieving high purity levels, which in turn directly influence the efficacy and safety of the nanoparticles in applications such as drug delivery or environmental remediation. The process of washing of the green synthesized NPs has the potential to remove the bioorganic capping layer. This has the consequence of hindering accurate analysis and interpretation of the resulting spectroscopic data. Research has demonstrated that the initial washing step is of particular significance, as it facilitates the removal of molecules that are only weakly bound to the surface of NPs. In contrast, the remainder of the biomacromolecular shell remains stable even after repeated washing [35]. Incomplete removal of precursors, especially metal ion precursors (e.g., acetates, nitrates) can also affect the quality of the FTIR spectrum as their residues may be visible in the FTIR spectra.

FTIR spectra of complex biological materials or mixtures often exhibit overlapping peaks. This complicates the identification of specific functional groups and can lead to misinterpretation of the data. The use of second derivatives of the spectra can be a fruitful approach for the purpose of revealing unresolved bands, particularly in the context of complex spectra derived from biological samples [35,37,38]. Implementing machine learning algorithms to analyze FTIR spectra can help in deconvoluting complex spectra and identifying overlapping peaks that may obscure important information about the NPs functional groups [39,40].

The utilization of chemometric techniques such as principal component analysis (PCA) and partial least squares (PLS) modelling has improved the interpretation of complex FTIR spectra. These methods facilitate enhanced classification and quantification of spectral data, thereby simplifying the analysis of the chemical composition of nanoparticles. The utilization of PCA and linear discriminant analysis (LDA) enables the clear revelation of discrepancies between the obtained spectra [41]. Nevertheless, the development of standardized methodologies for the preparation of biological samples, as well as the mathematical methods for the analysis of the resulting complex spectra, remains ongoing [35].

The employment of novel approaches and techniques has enabled researchers to circumvent the limitations associated with FTIR in the assessment of green nanoparticles. This has resulted in more accurate characterization and enhanced understanding of their properties and potential applications in fields such as medicine and environmental science. As previously stated, combining FTIR with other techniques such as UV–Vis spectrophotometry, DLS, TEM, EDX and XRD can provide a more comprehensive understanding of NPs properties. Combining FTIR with Raman spectroscopy can provide complementary information about molecular vibrations and chemical bonding [36]. This dual approach can help mitigate some limitations of FTIR, such as its inability to analyze non–polar compounds effectively [42,43]. The nano–FTIR spectroscopy technique combines FTIR with Atomic Force Microscopy (AFM) to achieve nanoscale resolution. It enables the characterization of materials at the molecular level, providing detailed information about the chemical composition and structure of NPs [18]. It is also important to consider other spectroscopic techniques that are useful for the analysis of green synthesized nanoparticles: near–infrared spectroscopy (NIR) and far–infrared spectroscopy (FIR).

In comparison to FTIR (operating within the mid–IR range), the NIR spectroscopy is more beneficial for a quantitative analysis of aqueous samples due to the significantly weaker nature of O–H absorption bands. The absorption bands in the near–infrared (wavenumber range from ~13,000 to 4000 cm^−1^) are frequently overtones and combination bands of group frequencies (4000–1500 cm^−1^ within mid–IR range, i.e., O–H, C–H and N–H group vibrations). NIR spectroscopy may be useful for the investigation of nanostructured materials such as nanoporous silica particles, dendrimers, nanocoated capillaries and carbon nanomaterials [44,45]. FIR spectroscopy is a technique that involves the absorption of infrared radiation in the far–infrared region, typically between 400 and 10 cm^−1^. It is used for studying lattice vibrations in samples and finds applications in the analysis of a variety of materials, including metals, metal oxides, metal sulphides and metal–ligand complexes [46].

## 3. The Application of FTIR Spectroscopy to Green Synthesized Nanoparticles Characterization

**Figure 2:**
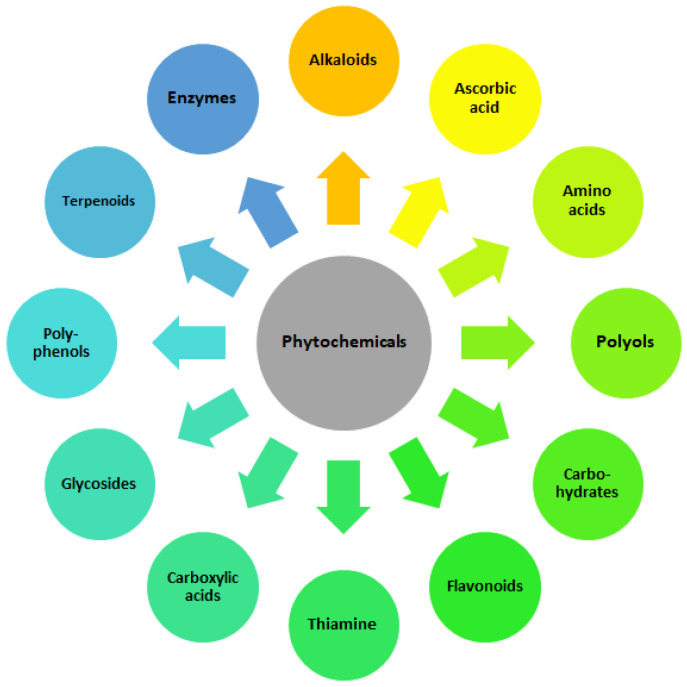
Important bioreductants found in plant extracts.

The distinctive characteristics of NPs are typically determined by the synthesis method employed. Even minor alterations in the technological process can result in significant variations in their intrinsic properties. Successful green synthesis of nanoparticles relies on a combination of biological agents (microorganisms or plant extracts), metal precursors, optimized reaction conditions (pH, temperature) and process optimization strategies. The morphology of the NPs formed is dependent on the type of plant extract and its concentration. The temperature and pH of the extract medium exert control over the growth and size of the NPs [47]. The most important role of biological agent is to act as reducing, capping and stabilizing factor. Reducing agents are substances that facilitate the reduction of metal ions to their elemental form, which is essential for the formation of nanoparticles. The primary functional groups engaged in the reduction of metal ions are the hydroxyl, carbonyl, amino and methoxide groups, which interact with the metal ions via electrostatic forces, resulting in their reduction [48]. In green synthesis, natural materials such as plant extracts or biological molecules, such as polyphenols [49], glycosides [50], carbohydrates [51], amino acids [52], terpenoids, carboxylic acids and flavonoids [53], polyols [54], enzymes [55], alkaloids [56], thiamine [57], ascorbic acid [58], having in their molecular structure the mentioned functional groups, often serve as reducing agents [59] (Figure 2). For example, phenolic compounds in plant extracts can reduce silver ions (Ag^+^) to silver nanoparticles (Ag^0^) while also providing some stabilization [59,60,61]. Enzymes produced by microorganisms can also function as bioreductants [55,62], as well as polysaccharides isolated from agricultural waste [63] and extracellular polymeric substances (EPS) produced by bacteria [64]. Reducing agents and capping agents may be added separately during physical and chemical methods of NPs synthesis. Capping agents are used to coat the surface of nanoparticles after they have formed. Their primary role is to prevent agglomeration and to stabilize the nanoparticles in a colloidal solution. Capping agents can influence the size, shape and overall properties of the nanoparticles, forming mono– or multi–layered protective coatings and providing long–term stability for the NPs. They often contain functional groups that bind to the nanoparticle surface, providing steric hindrance that prevents the particles from coming into close proximity and aggregating. They can also be used for biofunctionalization, i.e., to provide the presence of functional groups for attaching other biomolecules or drugs [65,66,67]. Capping agents can be broadly categorized into the following groups: organic ligands, surfactants, polysaccharides, proteins (i.a., bovine serum albumin, BSA), amino acids (i.a., ethylenediaminetetraacetic acid, EDTA, synthetic amino acid), lipids, polymers (i.a., chitosan, polyethylene glycol, PEG, polyvinylpyrrolidone, PVP), dendrimers, cyclodextrins and bio–extracts [65]. The differences between the aforementioned entities can be attributed to their functional groups and surface charge. The selection of capping agents is dependent on the targeted size and morphology of NPs, in addition to their potential applicability. Other factors are also taken into consideration, including the type of functional groups, the hydrophobic or hydrophilic nature of the functional groups and the charge on capping agents, the length of carbon chains/linkers and so forth. A slight modification in any particular parameter can have a considerable impact on the overall NPs properties.

**Figure 3:**
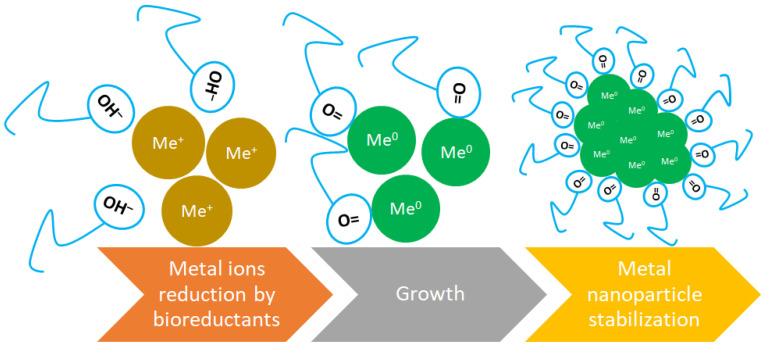
Green synthesis mechanism (based on [70]).

While sometimes used interchangeably with capping agents, stabilizing agents specifically refer to substances that maintain the stability of nanoparticles in suspension over time. This includes preventing sedimentation and ensuring that the nanoparticles do not aggregate during storage or application. Stabilizing agents can be capping agents but may also include other types of molecules that do not directly bind to the nanoparticle surface [68,69]. In the context of green synthesis of nanoparticles, reducing agents, capping agents and stabilizing agents are not the same, although they can sometimes overlap in function. The main mechanism of green biosynthesis through plants is illustrated in Figure 3.

FTIR analysis can be employed to identify the functional groups of diverse active substances or plant secondary metabolites that are accountable for the reduction, capping and stabilization of NPs. FTIR can be therefore successfully used in the analysis by (1) assessing the chemical identity of nanoparticles synthesized through various methods, including green synthesis techniques that utilize bio–based materials, (2) identification of functional groups accountable for the reduction of metal ions to their elemental forms, (3) identification of functional groups, thus revealing the presence of biomolecules that act as capping agents, which are crucial for stabilizing the nanoparticles by keeping them separated, eluding the aggregation and coalescence and (4) optimization of synthesis conditions by analyzing the FTIR spectra at different time intervals, which can allow one to determine how reaction conditions affect the formation and characteristics of nanoparticles, such as size and stability [71,72].

In the synthesis of green nanoparticles, various functional groups of secondary plants metabolites play a role in the reduction and stabilization of NPs. These include, among others, phenolic compounds (i.e., phenolic acids), terpenoids, saponins, flavonoids, steroids, alkaloids, tannins, proteins and peptides, sugars and polysaccharides. A number of these metabolites are capable of acting as both reducing and stabilizing agents, thereby inhibiting the aggregation and agglomeration processes. The reduction potential of ions and the reducing capacity of plants are influenced by the presence of polyphenols, enzymes and other chelating agents, which are intrinsic to plants. Phenolic –OH groups are prevalent in polyphenols and are known for their strong reducing capabilities. Phenolic compounds, possessing hydroxyl and carboxyl groups, are one of the best candidates for nanoparticle synthesis, and their antioxidant action is due to their high tendency to chelate metals [73], facilitating their reduction to nanoparticles. The phenolic compounds, especially those from the polyphenol family, often act as electron donors in the reduction process, making them vital in the synthesis of nanoparticles [8,12]. The nucleophilic aromatic rings of such compounds provide substantial support for antioxidant activity and metal chelation. In the presence of elevated levels of polyphenols, a protective coating was observed around the nascent nanoparticles, which inhibited their coalescence and aggregation. Terpenoids, which include many essential oils, can stabilize nanoparticles and enhance their formation through redox reactions, contributing to the overall efficiency of the synthesis process [73]. Based on the FTIR spectroscopy data, it was suggested that dissociation of a proton of the eugenol –OH groups results in the formation of resonance structures capable of further oxidation. This process is accompanied by the active reduction of metal ions, followed by nanoparticle formation [74]. Alkaloids, nitrogen–containing compounds, can also participate in the reduction of metal ions. Proteins found in plant extracts can reduce metal ions through mechanisms involving electron transfer. Amino acids, i.e., tryptophan and tyrosine, enhance the reduction process due to their specific side chains that can donate electrons [75,76]. Amino groups in proteins and amino acids can bind to metal surfaces, providing steric hindrance that stabilizes the nanoparticles and prevents their aggregation [67]. Carboxyl groups (–COOH) found in organic acids and amino acids, can interact with metal ions, enhancing stabilization through electrostatic repulsion and forming complexes that prevent particle aggregation [77]. Carbohydrates can stabilize nanoparticles during synthesis by providing steric stabilization through their large molecular structures, which create a physical barrier around the nanoparticles and may also contribute to the reduction of metal ions by acting as reducing agents themselves [78].

In FTIR spectra, the position of functional groups involved in reduction, capping and stabilization of metallic nanoparticles produced with plant extracts, exhibit a high degree of consistency, irrespective of the metal ions employed as the precursor for the NPs. Therefore, this review will focus on the most frequently described metal and metal oxide nanoparticles.

### 3.1. Metal Nanoparticles

As previously stated, metal nanoparticles themselves have no infrared absorption properties, thus direct FTIR analysis of pure nanoparticles is not possible. However, FTIR spectroscopy allows the identification of functional groups in the molecules under study, which in turn allows one to recognize the groups responsible for the reduction, capping and stabilizing of nanoparticles. A comparison of the spectrum of the pure extract used to obtain the NPs with that of the NPs synthesized using this extract may reveal the disappearance, reduction in intensity or shift of several characteristic peaks. This may indicate the involvement of these groups in the reduction process. Conversely, if the FTIR peaks remain unchanged, it suggests that these specific functional groups are responsible for the stabilization of the nanoparticles.

Given the importance of NPs shape and size in biomedical applications and toxicology, precise and controlled synthesis is essential. In order to enhance the biocompatibility of NPs, it is advisable to utilize non–toxic, green reagents. Literature reports indicate that the vast majority of antioxidants in bio–based extracts can serve as reducing, capping and stabilizing agents. Green synthesis was applied, i.a., to obtain metallic Au, Ag, Pt, Pd, Cu, Ti nanoparticles [79,80,81], flexible nanostructures with controllable shape, composition, size, structure and optical properties.

#### 3.1.1. Gold Nanoparticles

Gold nanoparticles (AuNPs) have garnered considerable interest across a multitude of disciplines, particularly in the biomedical realm. Their distinctive physical and chemical attributes, including their nanoscale dimensions, extensive surface area and tunable optical properties, render them well–suited for a wide range of applications, including diagnostics, therapy and drug delivery. AuNPs are extensively used in biosensors for detecting biomarkers associated with diseases such as cancer and cardiovascular disorders [82,83,84]. Commonly seen in home pregnancy tests, AuNPs facilitate rapid diagnostic tests by providing visual signals when they bind to target molecules [85]. Their optical properties enable colorimetric detection methods, which can indicate the presence of specific analytes. Additionally, AuNPs enhance imaging techniques like electron microscopy and dark–field microscopy, providing high contrast for biological samples [86]. In medical imaging, AuNPs serve as effective contrast agents in computed tomography (CT) scans [87]. They have a longer circulation time in the bloodstream compared to traditional iodine–based contrast agents, improving imaging quality and allowing better delineation of blood vessels [88]. AuNPs can be functionalized with drugs or therapeutic agents, allowing for targeted delivery to specific cells or tissues. Their high surface area allows for the attachment of multiple molecules, enhancing the efficacy of drug delivery systems [89]. AuNPs exhibit antibacterial properties and are being explored for use in coatings for medical devices to reduce infection rates [87,90,91]. In addition to their use in the field of medicine AuNPs are employed in environmental sensing applications to detect pollutants. This is due to their sensitivity to alterations in their optical properties when they interact with a range of substances [92]. The conventional method for synthesizing AuNPs involves two steps: firstly, a chemical reduction process is employed to extract the AuNPs, and secondly, stabilization methods are used to prevent further aggregation [93]. It is possible to synthesize AuNPs of varying shapes and sizes by modifying the synthesis methods and routes employed.

In a study conducted by Elia et al. [94], four distinct plant extracts of sage, lemon verbena, rose geranium and pomegranate (*Salvia officinalis*, *Lippia citriodora*, *Pelargonium graveolens* and *Punica granatum*, respectively) were employed as reducing and stabilizing agents in the preparation of AuNPs. The pure extracts and the resulting colloidal solutions of AuNPs were subjected to FTIR spectroscopy. The analysis of the spectra of the pure extracts enabled the identification of functional groups, and a change in the position of previously identified bands was observed in the AuNPs spectra. The band of hydroxyl groups (alcohols, phenols) and N–H (primary (1°) amines, amides) was identified at approximately 3330 cm^−1^, the band of P–H groups (phosphines) at 2332 cm^−1^, and the band of C=O groups at 1848 cm^−1^ and 1714 cm^−1^ was attributed to anhydrides and aldehydes/ketones, respectively. The N–H group bands were identified at approximately 1613 cm^−1^ (1°, secondary (2°) amines) and in the range 1606–1574 cm^−1^ (1° amines). The band of C=C groups in aromatic compounds was observed at 1420 cm^−1^. The band at ~1270 cm^−1^ was attributed to the vibrations of the S=O groups (sulfones, sulfonates), C–N groups (aromatic amines) and C–O groups (alcohols, carboxylic acids, esters, ethers). The band at approximately 1109 cm^−1^ was attributed to the vibrations of the C–N groups (aliphatic amines). In the AuNPs spectra, these bands were shifted towards higher or lower wavenumbers or completely disappeared. Authors hypothesized that these shifts in peaks positions were attributable to the adsorption of extract constituents onto the AuNPs surface.

The bioactive compounds present in the leaf extract of olive (*Olea europaea*) were engaged in the synthesis of AuNPs, which were subsequently identified through FTIR spectroscopy [95]. FTIR results demonstrated the presence of O–H (3409 cm^−1^) and C=O (1733 cm^−1^) stretching possibly of oleuropein, apigenin–7–glucoside and/or luteolin–7–glucoside, C–C stretching vibrations and –C–O–H bands at ~800 cm^−1^ and 1261 cm^−1^, respectively, in AuNPs. Additionally, a sharp, strong band observed at 1077 cm^−1^ was assigned to the C–OH groups of the proteins in *Olea europaea* extract. The shift of C=O stretching to 1721 cm^−1^ suggested that the biomolecules were bound to the AuNPs through this group. The band at 1624 cm^−1^, which has been assigned to amide I, has become more prominent in the spectrum of AuNPs and split into two bands at 1622 and 1648 cm^−1^. The observed split is likely attributable to alterations in the secondary protein structure [96,97], which may arise from either processing–related modifications or interactions with the NPs. The presence of IR bands resulting from C=O stretching vibrations in AuNPs spectrum and the emergence of amide I bands with a shift from that of the plain leaf suggested that proteins and antioxidant molecules bind to gold nanoparticles via free amine, C=O and O–H groups. This confirmed that the phytochemicals present in the extract acted as a reducing and stabilizing agent. Suriyakala et al. [98] performed FTIR spectra which suggested that phenolic compounds from peregrina (*Jatropha integerrima Jacq.*) flower extract were likely the agents responsible for the reduction of gold ions and formation of AuNPs. The presence of phenolic compounds was indicated by the peaks at 3237 cm^−1^ (phenolic O–H stretching) and 760 cm^−1^ (out of plane deformation of O–H group). Other bands indicate mainly protein content: 2920 cm^−1^ (C–H stretching), 1613 cm^−1^ (amine groups or carboxylate ion of the amino acid residues) and a band of C–N stretching in proteins at 1317 cm^−1^, demonstrating that protein acts as a ligand for AuNPs.

Ethanolic extract from gotu cola (*Centella asiatica*) leaves was used for the synthesis of AuNPs [99]. High content of phenolic compounds with strong antioxidant activity in the leaf extract was instrumental in reducing Au^3+^ to Au^0^ and the presence of phenolic groups was confirmed while analyzing FTIR spectrum of *C. asiatica* extract. The spectrum of extract revealed bands characteristic for alcohol (3380 cm^−1^ and 911 cm^−1^), phenolic groups (1130 cm^−1^), C–N stretching vibration of aromatic and aliphatic amines (1376 cm^−1^ and 1050 cm^−1^, respectively), methyl group deformation vibrations (1432 cm^−1^), C=C in aromatic structures (1607 cm^−1^), carbonyl (1695 cm^−1^) and C–H groups stretching (2850 cm^−1^). Additionally, FTIR spectrum of AuNPs exhibited bands, indicating that some phenolic compounds were bound to the surfaces of AuNPs, which remained despite repeated washing. These bands originated from the functional groups (alcohol, amines, phenols, carbonyl) present in the various plant metabolites of the leaf extract. Spectroscopic results revealed that high stability of nanoparticles was achieved due to the presence of amino (–NH_2_) or carboxylic (–COOH) groups.

Babu et al. [100] synthesized AuNPs using water hyssop (*Bacopa monnieri*) ethanolic extract. The authors assigned the bands in the FTIR spectrum of AuNPs to O–H hydroxyl groups (3394 cm^−1^), amide I in proteins (1632 cm^−1^), C–H groups vibration (1377 cm^−1^) and C–N stretching in amide groups (1085 cm^−1^), and indicated that these functional groups facilitated the reduction of the Au^3+^ ions to Au^0^ and capped the AuNPs during the particle growth termination process. AuNPs were synthesized using dragonhead (*Dracocephalum kotschyi*) leaf extract [101]. FTIR analysis identified the biomolecules which were involved in nanoparticles synthesis. IR spectrum of aqueous leaf extract revealed broad band at 3387 cm^−1^ (hydroxyl groups of alcohols or N–H of amines), a band at 2923 cm^−1^ (stretching of C–H groups), a band at 1416 cm^−1^ (C–N stretching vibration of aromatic amines) and a band at 1603 cm^−1^ (carbonyl group of carboxylic acid or amides). In turn, the FTIR spectrum of AuNPs revealed a slight shift in the N–H and/or OH stretching band from 3387 cm^−1^ to 3385 cm^−1^, C–H band from 2927 cm^−1^ to 2919 cm^−1^, C=O stretching from 1603 cm^−1^ to 1650 cm^−1^, C–N stretching from 1417 to 1441 cm^−1^ and –C–O–C stretching from 1078 to 1026 cm^−1^. Bands of C–O–H bending and C–C stretching at 1261 and 800 cm^−1^, respectively, had higher intensity in the spectrum of AuNPs. FTIR spectra analysis confirmed the presence of groups, which may be accountable for the bioreduction of Au^3+^ ions, leading to the synthesis, stabilization and avoiding agglomeration of Au^0^ nanoparticles. Therefore, the components of *D. kotschyi* act as both bioreductants and surfactants. FTIR measurement was also carried out to study the interaction of the nanoparticles and to identify the possible biomolecules in mango (*Mangifera indica*) leaf water extract, which can be responsible for capping and stabilization of the AuNPs [102]. It was assumed that water soluble compounds such as flavonoids, terpenoids and thiamine are the capping ligands of the AuNPs. This was evidenced by the following bands in the IR spectra: stretching vibrations of the N–H and O–H groups (3273 cm^−1^), C=O and C=C (1737 and 1624 cm^−1^, respectively), –C–O and –C–O–C stretching (1369 and 1020 cm^−1^, respectively), C–N stretching of aromatic amine groups (1444 cm^−1^) and –C–O–H bending (1216 cm^−1^).

FTIR spectroscopy was employed for identification the reducing and capping agents of date palm (*Phoenix dactylifera*) water extract and to understand the reaction mechanism between the components of the extract and gold salt [103]. Analysis of FTIR spectra revealed the presence of an intensive band at 3415 cm^−1^ (–OH groups), which shifted to 3398 cm^−1^ and became broader upon interaction with gold salt. Stretching vibrations at 1632 cm^−1^ of the C=O groups (most probably amide I band), visible in the extract spectrum, split into two peaks at 1651 and 1613 cm^−1^ in the AuNPs spectrum, which indicate a change in the secondary structure of proteins [98,99]. Thus, –OH groups and/or C=O groups present in carbohydrates, flavonoids, tannins, proteins and phenolic acids in *P. dactylifera* extract were accountable for the formation of AuNPs (coordination between Au^3+^ ions and the oxygen atoms of –OH and/or C=O).

AuNPs were prepared using the extract of lemon juice at different molar ratios as a reducing and capping agent [104]. Peaks of the –OH groups stretching vibrations were identified in FTIR spectra at 3433 cm^−1^, as well as C–H asymmetric and symmetric stretching vibration (2924 cm^−1^ and 2840 cm^−1^), C=O stretching of amide I at 1633 cm^−1^ and C–C and/or C–O stretching vibration at 1047 cm^−1^. The band at ~612 cm^−1^ was attributed to Au–O stretching vibrations. The theoretical calculation indicating the position of the Au–O bond vibration peak in the IR spectrum was 720 cm^−1^, but the actual position in the spectrum at 612 cm^−1^ authors interpreted as a consequence of the electrostatic coordination of gold nanoparticles with an oxygen atom in the C=O group and with other functional groups in the lemon extract.

Banana fruit waste (BFW) as multi–functional reducing, capping and stabilizing agent was used for the synthesis of the AuNPs of diverse shapes [105]. FTIR spectroscopy was used for identification of the functional groups causing reduction, capping and stabilization of nanoparticles. Authors observed shift of bands in the spectrum of BWF extract in relation to bands in the AuNPs spectrum. The band of hydroxyl groups shifted from 3117 cm^−1^ to 3126 cm^−1^, the band of asymmetric stretching of C–H groups shifted from 2938 cm^−1^ to 2942 cm^−1^ and new peak of symmetric stretching of C–H groups at 2890 cm^−1^ appeared, while the one at 1341 cm^−1^ vanished (C–H deformation vibration). The peak of C=O stretching vibrations of amide I in proteins at 1678 cm^−1^ shifted to 1684 cm^−1^ and its intensity decreased. Authors suggested that the proteins were engaged in the active reduction of metal ions by oxidizing aldehyde to carboxylic acid. Another change was observed for C–O and C–C band, which shifted from 1056 cm^−1^ to 1063 cm^−1^. The modified peaks in AuNPs spectrum indicated that the nanoparticles were formed as a result of the BFW extract application. A comparison of the absorption bands of AuNPs revealed an increase in wavenumbers, which might be attributed to the formation of coordination bonds between AuNPs and functional groups (O–H, C=O) of carbohydrates, which are predominantly present in the BFW extract. Thus, it was demonstrated that BFW extract was capable of acting as a reducer, stabilizer and capping agent.

#### 3.1.2. Silver Nanoparticles

In recent years, silver nanoparticles (AgNPs) have become one of the most studied and explored nanostructures derived from nanotechnology, as nanosilver–based materials have shown interesting, challenging and promising properties suitable for various biomedical applications. AgNPs, exhibiting broad–spectrum antimicrobial activity against bacteria, viruses and fungi, are mainly used for antimicrobial and anticancer therapy, as well as to promote wound repair and bone healing, or as vaccine adjuvants, antidiabetic agents and biosensors [106,107]. They serve as carriers for drugs, enhancing bioavailability and targeting specific tissues or cells. This targeted delivery system is particularly beneficial in cancer therapy, where AgNPs can deliver anticancer drugs directly to tumour cells [108]. AgNPs are also employed, i.a., in the field of imaging and diagnostics, catalysis and sensing, environmental remediation, electronics and optoelectronics and food industry [109].

As it was mentioned, FTIR spectroscopy may be used to probe the chemical composition of the surface of nanoparticles. FTIR spectroscopy was employed to investigate the chemical composition of the surface of the AgNPs prepared using aqueous seed extract of horsegram (*Macrotyloma uniflorum*) [110]. The strong and intense band of C=O stretching in –COOH groups of reducing agent was observed at 1721 cm^−1^. The band at 1614 cm^−1^ was attributed to the amide I band, while the amide II band manifested as a shoulder at 1574 cm^−1^. The occurrence of the amide bands was due to the carbonyl stretch (amide I) and C–N stretching and N–H bending (amide II) [111] vibrations in the amide linkage of proteins present in the sample. The C–O–H groups vibrations was visible at 1443 cm^−1^ and the band at 1222 cm^−1^ was assigned to polyphenols, whereas the band at 1074 cm^−1^ corresponded to C–N stretching vibrations of aliphatic amines. The presence of compounds having these functional groups indicated a dual function of reduction and stabilization of AgNPs, namely the involvement of proteins in capping and polyphenols in reduction of Ag^+^ ions. Proteins may therefore play a role in the biosynthesis and stability of metallic nanoparticles.

The green biosynthesis of AgNPs using the plant extract of sage (*Salvia spinosa*) demonstrated that compounds containing –OH and C=O groups played a role in the reduction and stabilization of AgNPs [112]. Analysis of FTIR spectra revealed the presence of phenols and alcohols with free OH group and/or N–H stretching vibrations (~3423 cm^−1^), both in extract and AgNPs spectra. The band observed at ~2359 cm^−1^ indicated the presence of symmetric stretching of COO^–^; however, this was only evident in the spectrum of AgNPs. Analysis of spectra also confirmed the presence of compounds with: C≡C groups (2240 cm^−1^), N–C and N=C groups in R–N=C=S structure (2138 cm^−1^), C=C in aromatic structures and/or amide I (1630 cm^−1^), amide II in proteins (~1520 cm^−1^), S=O (sulphate ester) groups (1407 cm^−1^), C–O in COOH (~1150 cm^−1^), =CH in aromatic structures (821 cm^−1^, 675 cm^−1^), C–Cl (alkyl halides) (603 cm^−1^). The authors attributed slight shifts in peak positions to the involvement of plant extract compounds in the biosynthesis of AgNPs.

The –C=O groups of amino acids often function as capping ligands for NPs, and FTIR spectra allow one to confirm the involvement of diverse C=O groups on the NP surface, which prevents their aggregation and maintains their stability in the aqueous phase. Proteins can bind to AgNPs through free amine groups in the proteins [113] and stabilize them by surface–bound proteins [114]. Cell free, rich in proteins and enzymes extract of fungus *Candida albicans* was used to prepare gold and silver nanoparticles [115]. The presence of proteins was confirmed by the bands at 3225 cm^−1^ (–NH_2_ in primary amides), ∼1656 cm^−1^ and 1520 cm^−1^ (C=O stretching and N–H bending of amide groups in proteins, respectively), bands at 1371 cm^−1^ (AuNPs) and 1380 cm^−1^ (AgNPs) due to C–O stretching of the carboxylate ions in amino acids. Other bands were an indication of the presence of the nitrogen compounds like nitriles (–C≡N) and cyanates (–O–CN) (2355 cm^−1^), and C–H stretching at 2850 cm^−1^. The peak at ∼509 cm^−1^ was attributed to the metal–ligand stretching resulting from the interaction of biomolecules with the nanoparticles surfaces. The findings of the FTIR investigation have validated the hypothesis that the carbonyl group present in amino acid residues and peptides of proteins has the capacity to form stronger binding interactions with metallic elements. The resulting complexes were observed to encapsulate AuNPs and AgNPs, effectively preventing agglomeration and stabilizing the nanoparticles in their surrounding medium.

Wan Mat Khalir et al. [116] biosynthesized AgNPs mediated by aqueous stem extract of *Entada spiralis*, containing terpenoid saponins and glycosides, which are able to act as a reducing agent due to the presence of carboxyl and hydroxyl groups. When analyzing the spectra of the *E. spiralis* extract and synthesized with the extract AgNPs, some shifts in the bands and increase/decrease in peaks intensities were noticeable, suggesting that following functional groups were involved in the binding mechanism on the AgNPs: the peaks observed at 3399 cm^−1^ (hydroxyl O–H and/or amine –NH_2_), 2932 cm^−1^ (C–H stretching), 1522 cm^−1^ (aromatic ring of the terpenoid saponin structure) and 530 cm^−1^ (bonding of oxygen from the hydroxyl groups) exhibited a shift to 3402, 2925 cm^−1^, 1516 cm^−1^ and 521 cm^−1^ (Ag–O), respectively. The emergence of a new peak at 1829 cm^−1^ indicated the presence of carboxylate and C=C in the aromatic groups derived from the terpenoid saponin structure. This observation substantiated the hypothesis that the glucose moiety attached to the terpenoid saponin as an aldehyde underwent oxidation to gluconic acid. Additionally, the new peak at 823 cm^−1^ appeared, implying the potential bonding of C–H groups with the AgNPs. The authors also observed disappearance of bands at 1445 cm^−1^ and 1258 cm^−1^, which they attributed to vibrations of carboxylate groups and C–C(=O)–O stretching of the ester, respectively. The involvement of N–H groups of glycoside in the synthesis and stabilization of nanoparticles was confirmed by the decrease in the intensity of the peak at 1621 cm^−1^ in the spectrum of AgNPs. The band at 1072 cm^−1^ (C–O stretching) remained unchanged.

In a subsequent study, FTIR spectroscopy was used to identify compounds present in monarch redstem (*Ammannia baccifera*) leaf extract possibly responsible for reducing Ag^+^ into silver nanoparticles (Ag^0^). Based on the analysis of the spectra, Alam et al. [117] concluded that shifts or decreases in intensity of the specific peaks indicated the involvement of the following functional groups in the synthesis of AgNPs: –OH groups in alcohols and phenolic compounds (shift from 3266 cm^−1^ to 3323 cm^−1^ and from 1046 cm^−1^ to 993 cm^−1^ in AgNPs spectrum) and C=O stretching at ~1600 cm^−1^ (decrease in intensity). The alternation of these peaks indicated that water–soluble plant metabolites, including quinones, flavonoids and polyphenols, were involved in the synthesis of AgNPs.

The synthesis and characterization of green tea leaf extract–mediated AgNPs were conducted with a view to evaluating their biological activity and potential chemotherapeutic properties [118]. The objective of FTIR analysis was to investigate the functional groups in aqueous plant extracts in order to ascertain their roles, to determine how these groups facilitate the reduction of metal precursors into nanostructures and furthermore, to identify how these groups act as capping and stabilizing agents for the final end products. It appeared that the spectra of the extract and AgNPs exhibited a consistent pattern, with only minor variations in peak position and intensity, what indicated that these biomolecules were involved in the reduction of Ag^+^ and the stabilization of AgNPs. Some peaks in FTIR spectra shifted or their intensity changed, namely 3310 cm^−1^ in the extract spectrum (stretching vibration of the –OH group in the flavonoid ring’s phenolic component) shifted to 3315 cm^−1^ in AgNPs spectrum, intensity of peaks at 2920 and 2852 cm^−1^ (stretching vibrations of the C–H groups of aliphatic hydrocarbons) decreased, and those observed at 1145 cm^−1^ (C–O–C in carbohydrates), at 1029 cm^−1^ (C–N stretching) and in the 822–722 cm^−1^ range (C–H and/or C–S) shifted to 1183, 1092 and 789–787 cm^−1^ in the green synthesized AgNPs, respectively. The other peaks indicated the presence of the following functional groups: C=O stretching of polysaccharide components and proteins’ primary and secondary amide bonds (1624 cm^−1^), =NH (1440 cm^−1^), C–O–H bending of alcohol (1368 and 1250 cm^−1^) and C–N stretching (1029 cm^−1^). The peak at ~600 cm^−1^ pointed to the formation of Ag–O. Other authors also indicate the presence of Ag–O–Ag stretching vibrations and the appearance of this peak while formation of silver nanostructures [119,120]. FTIR analysis revealed that the diverse range of compounds present in the aqueous green tea leaf extract, including proteins, polysaccharides, flavonoids and primary and secondary amines, facilitated the reduction of Ag^+^ to generate AgNPs. The presence of functional groups, namely free amine groups from proteins and carbonyl groups from flavonoids, played a crucial role in stabilizing and capping the resulting silver nanoparticles.

FTIR spectroscopy was used to reveal the functional groups involved in the surface coating and stabilization of the AgNPs synthesized with aqueous and hydroalcoholic extracts of tansy flowers and leaves (*Tanaceti flos* and *Tanaceti folium*, respectively) [121]. Extracts of tansy contain essential oils with varying compositions, including beta–thujone, isomers, terpenes and camphene. Additionally, the essential oils contain various other compounds, including monoterpenes (such as alpha–pinene, beta–pinene and myrcene), polyphenols, amino acids, proteins, flavonoids (derivatives of quercetin and luteolin), oxygenated monoterpenes, monoterpene hydrocarbons, alcohols, carbohydrates, enzymes and mineral compounds. The observed shift of some peaks and the emergence of new peaks in the spectra of AgNPs substantiate the bioreduction of Ag^+^ to Ag^0^ by specific functional groups of plant extract: a shift of the phenolic groups bands from ~1395 cm^−1^ to 1441 cm^−1^ and 1258 to ~1240 cm^−1^, a shift of the ether groups bands from 1060 cm^−1^ to 1025 cm^−1^ (*Tanaceti flos* extract) and from 1045 cm^−1^ to 1029 cm^−1^ (*Tanaceti folium*) and disappearance of C–CO–C and C–CO in–plane deformation vibrations in ketone groups (~598 cm^−1^ and ~510 cm^−1^). The spectra of AgNPs exhibited the presence of COOH carboxyl groups bands (~3280 cm^−1^ and 1731 cm^−1^). These bands were a consequence of the oxidation of phenolic groups. A slight shift or decrease in the intensity of the aforementioned bands, accompanied by an increase in the intensity of the amide II band at ~1520 cm^−1^ and the amine C–N stretching vibrations at 1027 cm^−1^ in the AgNPs spectra, suggested that proteins extracted from tansy coated the silver nanoparticles and stabilized them. High intensity of the amide groups band at 1516 cm^−1^ also indicated the coverage of the AgNPs with those groups and their stabilization. The analysis of the spectra also allowed the presence of ketones, aldehydes, quinones and esters (peaks between 1700 and 1600 cm^−1^) assigned to the C=O vibration of carbonyl groups and/or C=C vibrations of aromatic structures to be confirmed. The peaks at ~1595 cm^−1^ and ~1392 cm^−1^ in the extracts spectra also pointed to COO^–^ asymmetric and symmetric stretching, respectively.

Madaniyah et al. [122] explored the biosynthesis of silver nanoparticles using the medicinal plant Indian copperleaf (*Acalypha indica* Linn) as a mediator under sunlight and at 60 °C. The synthesized AgNPs were further coated by water–soluble chitosan to stabilize the nanoparticles in order to improve their anticancer efficacy. Authors observed shifts in FTIR spectra, which they attributed to the reduction and adsorption of bioactive functional groups from the extract onto the surface of AgNPs. Additionally, the presence of amide bands at 1556 cm^−1^ (N–H bending of the chitosan amide) in AgNPs with chitosan suggested that the interaction between AgNPs and chitosan did not disrupt the secondary structure during the synthesis process. The presence of strong peaks at ~470 cm^−1^ (C–O–Ag) suggested the formation of AgNPs via carbonyl bonds from flavonoids or alkaloids in *Acalypha indica* L.

AgNPs are the most widely used nanomaterial as a broad–spectrum antimicrobial agent, due to the release of silver ions from the crystalline core of these particles, which contributes to their antimicrobial toxicity [123]. However, bacterial adaptation and their defence mechanisms have the potential to compromise the efficacy of disinfection processes or mitigate unintended consequences, such as alterations to the microbial ecosystem services associated with silver release into the environment. Extracellular polymeric substances (EPSs), composed mostly of polysaccharides and proteins, secreted by bacteria could play a role in protecting cells against environmental stresses. As demonstrated by Kang et al. [64], *Escherichia coli*–secreted EPSs can reduce Ag^+^ and entrap it as AgNPs, thereby acting as a permeability barrier to impede intracellular penetration by silver. Authors have compared the FTIR spectra of an EPS before and after reaction with Ag^+^ to prove the involvement of reducing sugar components of EPS in Ag^+^ reduction. Following the reaction with Ag^+^, the bands of rhamnose (988 cm^−1^) and pyranose (924 cm^−1^), structures in FTIR spectra exhibited a significant decrease in intensity, while the band of carboxyl groups (1385 cm^−1^) demonstrated a notable enhancement in intensity and clarity. This observation allowed us to conclude that the aldehyde groups present in the reducing sugars had undergone oxidation to form carboxyl groups by Ag^+^. These results were confirmed by ^13^C nuclear magnetic resonance (^13^C NMR) analysis.

#### 3.1.3. Platinum Nanoparticles

Platinum nanoparticles (PtNPs) have garnered significant attention across various fields and their applications span diagnostics, catalysis and biomedical uses, showcasing their potential in both industrial and medical settings. PtNPs can be utilized in catalytic processes, including automotive catalytic converters, oxidation and hydrogenation processes, hydrogen fuel cells but also in electrochemical reactions and in sensors for detecting various substances [124,125]. The biomedical field has seen innovative uses of platinum nanoparticles due to their biocompatibility and reactivity. Key applications include cancer treatment, photothermal therapy, drug delivery systems, diagnostics and antibacterial agents [126].

Halfa grass (*Desmostachya bipinnata*) extract was used for preparation of platinum nanoparticles incorporated into mouthwash formulations [127]. The objective of FTIR study was to identify the functional groups present and to examine the interactions between the nanoparticles and biomolecules. Alkyl halides, visible in the spectrum as a peak at 688 cm^−1^, were responsible for the stability of PtNPs and preventing their aggregation. The peak of O–H stretching vibrations at ~3435 cm^−1^ indicated the presence of hydroxyl groups, which are essential for bioreduction as they donate electrons to reduce Pt^4+^ to Pt^0^, thereby initiating the formation of nanoparticles. The carbonyl C=O functional groups of amide I, identified at ~1637 cm^−1^, were also crucial for the reduction of platinum ions. They contributed to the stabilization of the reduced metal atoms and the promotion of nanoparticle growth through their electron–donating capacity. Similar findings were reported by Kora and Rastogi [128], who synthesized PtNPs using renewable, biodegradable plant exudate gum and gum olibanum (*Boswellia serrata*) as a reducing and stabilizing agent. Based on the shifts in the peaks in FTIR spectra of gum and nanoparticles, the authors assumed that the reduction of platinum ions occurs due to the presence of hydroxyl and carboxylate groups of the gum (shift of O–H groups band from 3395 to 3464 cm^−1^; shift of carboxylate groups band from 1420 to 1433 cm^−1^ and from 1385 to 1368 cm^−1^; shift of C−O band of carboxylic acids from 1261 to 1219 cm^−1^, respectively). The stabilization of the PtNPs was assured by the functional groups of proteins (disappearance of amide I band at 1651 cm^−1^, appearance of C=O band at 1740 cm^−1^ and shift of amide II band from 1605 to 1535 cm^−1^).

Green synthesis of biogenic PtNPs from *Prunella vulgaris* (herbaceous plant in the mint family) leaf extract was performed in order to achieve synthetic enzyme–mimics (artificial nanozymes) [129]. Synthetic enzyme–mimics offer a number of advantages over their natural counterparts. These include greater stability against deactivation in a variety of chemical conditions, ease of surface modification, lower synthesis costs, less sophisticated purification steps and a longer shelf life. FTIR spectroscopy was used to demonstrate the effectiveness of the *P. vulgaris* extract characterized by a high concentration of active compounds, including flavonoids, alkaloids, anthraquinones, terpenoids and polyphenolics, as an effective reducing and stabilizing agents. Spectroscopic analysis revealed that the intensities and peaks positions of all identified functional groups in the extract exhibited a reduction and shift at the following wavenumbers: 3520 cm^−1^ (O–H stretching of phenolic compounds and N–H stretching), 2936 cm^−1^ and 1399 cm^−1^ (C–H), 1710 cm^−1^ (C=O), 1580 cm^−1^ (C=C), 1081 cm^−1^ (C–O–C in flavonoids) and 1037 cm^−1^ (C–OH). The bands observed at 823 cm^−1^ and 680 cm^−1^ are indicative of the presence of =CH in aromatic compounds and the peak at 774 cm^−1^ peak is suggestive of the presence of alkyl halides in the extract. The latter peak was also observed by Hosny et al. [130].

Persimmon (*Diospyros kaki*) leaf extract was used as a reducing agent in the eco–friendly extracellular synthesis of PtNPs [131] and FTIR spectroscopy was utilized to identify the biomolecules responsible for reducing platinum ions and stabilizing the platinum nanoparticles formed, but only the spectrum of PtNPs was made. The spectrum revealed the presence of intense bands at 1692 cm^−1^ (C=O), 1613 cm^−1^ (C=C in aromatic rings), 1410 cm^−1^ (methyl groups), 1325 cm^−1^ (C–N stretching in aromatic amines), 1195 cm^−1^ (phenolic groups), 1033 (C–N stretching in aliphatic amines) and 924 cm^−1^ (alcohols). No peaks of amide I (~1640 cm^−1^) or amide II (~1540 cm^−1^) characteristic of proteins were found, what indicated that PtNPs synthesized with *D. kaki* extract were surrounded by plant metabolites, such as terpenoids, which possess functional groups of amines, alcohols, ketones, aldehydes and carboxylic acids.

Platinum nanoparticles can also be synthesized using fungal species such as *Neurospora crassa* [132] and *Fusarium oxysporum* [133], where proteins, amides, long chain fatty acids and polysaccharides were pointed as biological molecules responsible for capping and stabilizing agents of PtNPs along with bioreduction of platinum ions. *Penicillium pinophilum* cell–free filtrate was used as reducing and stabilizing agent for PtNPs production [134]. The PtNPs synthesized using the fungal filtrate were observed to remain non–aggregated, indicating that a capping agent was responsible for their stabilization. This hypothesis was subsequently confirmed by FTIR analysis. The primary peaks observed in the fungal cell–free filtrate spectrum were also evident in the spectra of biogenic PtNPs, although with diminished intensities and minor shifts. The peaks observed at ~3313 cm^−1^ in fungal filtrate spectrum and attributed to N–H stretching (1°, 2° amines or amides) shifted to 3381 cm^−1^ in PtNPs spectrum. The peak at 1602 cm^−1^ (N–H bending in amines) shifted to 1621 cm^−1^, respectively. The peaks assigned to C–N stretching in amines at 1022 cm^−1^ shifted to 1056 cm^−1^. Therefore, the change in intensity and peak shifts indicated the presence of compounds that could serve as the reducing, capping and stabilizing agents. Thirumurugan et al. [135] have attempted to synthesize and characterize platinum nanoparticles using neem leaf (*Azadirachta indica*) water extracts. FTIR spectrum revealed the presence of following functional groups: C=O (1728 cm^−1^, 1643 cm^−1^), C–N (1527 cm^−1^, 1219 cm^−1^) and C–H (1365 cm^−1^). FTIR analysis confirmed the presence of functional groups such as carbonyls, alkanes and aliphatic amines.

### 3.2. Metal Oxide Nanoparticles

As previously stated, the bands of organic functional groups that play a reducing, capping or stabilizing role in metal nanoparticles synthesis exhibit a similar position in the IR spectra, irrespective of the metallic surface of the NPs to which they are bound. However, the analysis of FTIR spectra of metal oxide nanoparticles presents a certain degree of difficulty, given that the metal–oxygen bond, in contrast to the metallic bond, is discernible in the infrared region. Bands of metal–oxygen vibrations can overlap with bands of organic functional groups derived from extracts. Usually metal–oxygen bond peaks reside in the 800–400 cm^−1^ range, but broad peaks of hydroxyl group –OH vibrational stretching within 3700–3100 cm^−1^ range (maximum at ~3450 cm^−1^) as well as –OH bending of water (moisture) at ~1640 cm^−1^ may also appear [136,137]. In the infrared spectra of metal oxide nanoparticles, bands originating from metal precursors, i.e., nitrates, may also be discernible. The nitrates may be visible in the spectra as bands at ~1760, 1620, 1430, 1390, 1290, 1050, 830 cm^−1^ [138]. Therefore, these should also be taken into account when interpreting the FTIR spectra of metal oxide NPs. Table 2 provides a summary of the spectral positions of the infrared absorption bands exhibited by the metal oxides that constitute the nanoparticles.

There is a rising trend in the number of scientific articles being published that address the biosynthesis of metal oxide nanoparticles as well as their various applications, usually as photocatalysts. Cobalt oxide plant–based nanoparticles (CoONPs) were synthesized using the grape Jumbo Muscadine (*Vitis rotundifolia*) [142]. ZnO nanoparticles were biosynthesized with *Hibiscus sabdariffa* extract [154]. Magnetic iron oxide nanoparticles (Fe_2_O_3_NPs) were synthesized using the fruit extract of *Cynometra ramiflora*, a by–product of the fruit’s natural ripening process [157]. Iron oxide FeONPs nanoparticles were synthesized using leaf extract of minnieroot (*Ruellia tuberosa*) [144]. Cupric oxide nanoparticles (CuONPs) were prepared via plant mediated green synthesis with butter tree (*Madhuca longifolia*) extract [143]. Nickel oxide (NiONPs) nanoparticles were prepared using chia seeds (*Salvia hispanica* L.) extract [148]. Biosynthesis of tin dioxide nanoparticles (SnO_2_NPs) was achieved using extracts of tea (*Camellia sinensis*) [149]. Zinc oxide nanoparticles (ZnONPs) were successfully synthesized via chemical and green, environmentally benign method from banana peel waste extract [158]. All of the aforementioned nanoparticles demonstrated activity in photocatalytic reactions.

Calcium oxide nanoparticles CaONPs have been synthesized utilizing a green precursor *Lala clam* seashells [140] and tested as catalyst material for developing the modified electrode sensor in electroanalytical platform for urea detection in milk. *Moringa oleifera* extract was used for the green synthesis of magnetic iron oxide (Fe_3_O_4_NPs) nanoparticles, potential candidates for being integrated in photovoltaic devices [159]. Consequently, the robust magnetic properties of iron oxide nanoparticles (FeONPs) render them valuable in a number of medical imaging techniques. Potential applications of FeONPs include magnetic resonance imaging (MRI), targeted drug delivery systems and environmental remediation for the removal of contaminants [160].

Titanium dioxide nanoparticles (TiO_2_NPs) are among the most extensively researched materials, due to their photocatalytic properties and chemical and thermal stability, which make them suitable for use in a wide range of industrial applications [146]. These nanoparticles can be engineered to deliver drugs, including anticancer agents and antibiotics, directly to target cells. This targeted delivery is facilitated by their small size and ability to be functionalised with specific biomolecules [161]. TiO_2_NPs function as photosensitizers, generating reactive oxygen species (ROS) upon exposure to UV light. This property is exploited in the treatment of malignant tumours and the inactivation of antibiotic–resistant bacteria [161]. Due to their photocatalytic properties, TiO_2_NPs can be used to coat medical devices and implants, providing antimicrobial surfaces that reduce infection rates associated with these devices [162]. They are effective in the photocatalytic degradation of organic pollutants in wastewater.

The synthesis of TiO_2_NPs has typically been conducted using chemical routes. However, there is a growing trend towards the utilization of environmentally friendly, green synthesis methods. Neem (*Azadirachta indica*) leaf extract has been used for the synthesis of TiO_2_NPs [153]. FTIR study revealed the presence of terpenoids, flavonoids and proteins considered responsible for the formation and stabilization of titanium dioxide nanoparticles. Analysis of the TiO_2_NPs spectrum confirmed the presence of –OH groups in alcohols and phenolic compounds, C=C groups of aromatic rings, C=O vibrations of carboxylic acid and Ti–O–Ti stretching vibration of titanium dioxide nanoparticles at ~710 cm^−1^. In turn, Dobrucka [150] identifies the presence of TiO_2_ at 431 cm^−1^. The available literature data indicate that TiO_2_ exhibits strong absorption bands within the 700–600 cm^−1^ and 525–460 cm^−1^ ranges [136,152,163]. Similar environmentally benign methodology in the TiO_2_NPs synthesis has been implemented utilizing the plant species *Sesbania grandiflora* [164]. FTIR spectra of TiO_2_NPs revealed the presence of bands, which the authors attributed to –NH stretching of amide II (3403 cm^−1^), C–H stretch of the flavonoids (2914 cm^−1^), Si–H stretch of organosilicon (2266 cm^−1^), amide groups (1626 cm^−1^), C–N stretching of the aromatic amide group (1389 cm^−1^) and C–OH stretching in secondary alcohols (1047 cm^−1^). Thus, the presence of flavonoids, organosilicon compounds, aromatic amine groups and secondary alcohols, which were present in the *S. grandiflora* leaf extract, could act as capping ligands for the TiO_2_NPs.

Zinc oxide nanoparticles (ZnONPs) possess distinctive semiconducting, optical and piezoelectric properties. Their applications extend across a range of sectors, including industry, biomedicine and cosmetics, thereby demonstrating their multifunctionality [165,166]. ZnONPs have seen a surge in use in the rubber, paint, coatings and cosmetics industries and have attracted considerable attention in biological applications due to their remarkable biocompatibility, economic viability and minimal toxicity. ZnONPs have demonstrated significant promise within the field of biomedicine, particularly in the context of anticancer and antibacterial applications. This is attributable to their ability to induce elevated levels of reactive oxygen species (ROS), the release of zinc ions and the subsequent initiation of cell apoptosis [167]. ZnONPs have been demonstrated to enhance the mechanical strength and antibacterial properties of dental composites and have been shown to minimize bacterial adhesion and enamel demineralisation when used to coat orthodontic appliances [168].

FTIR spectroscopy was used to determine the functional groups of compounds present in the *Deverra tortuosa* extract involved in the formation and stabilization of green synthesized ZnONPs [155]. Analysis of peaks in FTIR spectrum revealed the presence of 1° and 2° amines, characteristic of proteins/enzymes, which have been shown to stabilize ZnONPs by forming a coating, covering the metal nanoparticles and preventing the nanoparticles from agglomerating. The presence of flavonoids and other phenolics, confirmed by the presence of O–H and C–OH stretching vibrations (3434 cm^−1^ and 1117 cm^−1^, respectively) have been shown to assist in the formation of ZnONPs. The presence of additional peaks indicated the hexagonal structure of the synthesized ZnO, with the peaks at 878 cm^−1^ and 442 cm^−1^. In the study of Uysal et al. [156] polyphenolic compounds were recognized as substances supporting formation of ZnONPs. Additionally, the formation of ZnONPs was confirmed by the peaks at 889 cm^−1^ and 554 cm^−1^.

ZnNPs were synthesized using onion waste peel extract (*Allium cepa* L.) via the green synthesis approach [169]. The FTIR data demonstrated the presence of metabolites, including flavonoids, alkaloids, carboxylic acids and polyphenols, which were found to be linked to ZnONPs. It was observed that these substances, particularly flavonoids and phenolics, played a crucial role in the transformation of Zn^2+^ into ZnONPs. The presence of carboxylic groups was found to contribute to the stabilization of ZnONPs through interaction with the NP surface.

Iron can form several types of nanoparticles, primarily categorized as iron oxide nanoparticles (IONPs) and zero–valent iron nanoparticles (nZVI). Each type exhibits distinct properties and applications. Recent research has focused intensely on investigating the potential of nZVI for the removal of organic and inorganic pollutants. It has been demonstrated that both nZVI and IONPs function through a Fenton–like mechanism, generating reactive oxygen species (ROS) that facilitate the degradation of pollutants. The efficacy of these materials in the removal of organic contaminants has been well–documented [170,171,172]. The results have demonstrated that nZVI can be effective for the treatment of contaminated soil and groundwater due to its elevated surface area, elevated reaction rate and large number of active sites [173,174,175]. Nevertheless, owing to their high reactivity, nZVI are susceptible to rapid oxidation when exposed to air. This oxidation results in the formation of iron oxides, thereby significantly reducing the reactivity of NPs and limiting their effectiveness in remediation applications. The immediate formation of oxide layers upon exposure to oxygen can hinder their intended reactions with contaminants [176]. IONPs maintain their reactivity over time better than nZVI, which can suffer from rapid passivation due to oxidation. This stability enhances their effectiveness in long–term remediation strategies [177]. Green synthesized nZVI, differing in shape, morphology and size, reveal a complete coating of the iron surfaces by, e.g., proteins and polyphenols. This coating has been found to inhibit the oxidation of the nanoparticles. Furthermore, the NPs have been observed to be uniformly dispersed throughout the reaction medium, exhibiting no signs of agglomeration [178,179]. Thus, the use of biogenic materials not only reduces reliance on harmful chemicals but also enhances the stability of the nanoparticles due to the capping effect of phytochemicals, like polyphenols and flavonoids, present in the extracts [180]. This leads to better dispersion and diminished aggregation compared to chemically synthesized nZVI.

It has been established that both IONPs and nZVI fulfil key roles in a range of applications, yet it is widely accepted that the former are considered more favourable in biomedical contexts on account of their superior biocompatibility and multifunctionality. IONPs, particularly magnetite (Fe_3_O_4_), maghemite (γ–Fe_2_O_3_) and hematite (α–Fe_2_O_3_), are known for their biocompatibility and low toxicity, making them suitable for medical applications [181]. In addition to this, in environmental settings, IONPs performance in terms of pollutant degradation and stability is also enhanced relative to that of nZVI nanoparticles [182].

As mentioned, the potential applications of IONPs are manifold, extending across a number of disciplines. This demonstrates their potential as multifunctional agents in biomedical fields such as diagnostics and therapeutics. The distinctive magnetic properties, biocompatibility and ease of surface modification render them suitable for a wide range of innovative uses [181]. The green synthesis of IONPs generally yields amorphous iron oxide [178], which is considered to be the most unstable form among its diverse types [183]. However, it is important to form stable and crystalline IONPs for precise characterization and the potential utilization in a range of applications. The process of calcination may be a strategy to enhance the performance of iron oxide. The synthesis of IONPs with magnetic core followed by calcination was achieved through the utilization of clove and green coffee extracts [182]. Eugenol, as identified through FTIR analysis, functioned as a reducing and stabilizing agent for clove extract–assisted NPs, and polyphenolic acids (chlorogenic acids) and caffeine for coffee extract–assisted NPs were responsible for the reduction of Fe^3+^ to Fe. Following the annealing of IONPs at 550 °C, the authors identified bands at 597 and 462 cm^−1^ in the FTIR spectrum of clove extract–assisted NPs, and 549 and 460 cm^−1^ in the spectrum of coffee extract–assisted IONPs, which they attributed to Fe–O bonds. XRD was employed to confirm magnetite and magnetite/hematite formation (clove extract–assisted NPs and coffee extract–assisted NPs, respectively). It is noteworthy that the position of the Fe–O bands in the FTIR spectrum of the Fe_2_O_3_ is contingent on parameters such as particle size, shape and structure [184]. Consequently, the observed variation in the position of the bands may be attributed to these factors.

In the study of Abdullah et al. [185], IONPs in the form of magnetite and maghemite were synthesized using date palm (*Pheonix dactylifera*) leaf extract. The analysis of FTIR spectra indicated that phenolic groups were responsible for the reduction of Fe^3+^ ions. The band of phenolic O–H stretching shifted from 3416 cm^−1^ in *P. dactylifera* leaf extract spectrum to 3375 cm^−1^ in IONPs spectrum. Additionally, the reduction of Fe^3+^ into IONPs caused the 1643 cm^−1^ IR band of *P. dactylifera* to be split into multiple peaks at 1653, 1633 and 1623 cm^−1^. The authors hypothesized that this was due to the coordination of Fe^3+^, Fe^2+^ ions and the oxygen atoms of –OH and/or C=C groups during the formation of IONPs. The authors also employed FTIR to evaluate the proportion of magnetite and maghemite in the NPs formed, although they noted that FTIR was unable to accurately quantify the proportion of each phase. FTIR spectra of the synthesized IONPs exhibited peaks that were characteristic of both magnetite (a broad band with a maximum at 573 cm^−1^, accompanied by a shoulder at ~700 cm^−1^) and maghemite (six bands within the 800–500 cm^−1^ range, with the strongest band occurring at 638 cm^−1^).

To summarize, the principal wavenumber ranges of functional groups engaged in the reduction, capping and stabilization of nanoparticles, as documented in the literature, are presented in Table 3.

The analysis of peaks in FTIR spectra, their spectral shifts and intensities variations can provide insights into the reduction of metal ions and other structural alterations that are associated with the synthesis and stabilization of nanoparticles. The reduction of metal ions to NPs during the synthesis process and their further stabilization is indicated by changes, i.a., in O–H, C=O, –COOH, C–O–C, N–H, C–N bands position and intensity. The bands of O–H groups shift towards lower or higher wavenumbers, depending on the presence of hydrogen bonding. If a hydrogen bonding is formed, the band shifts to lower wavenumbers [186]. When the O–H group is isolated, e.g., because of steric hindrance effects, the band is narrower and observed at higher wavenumbers. Additionally, the loss of hydroxyl groups, e.g., the consumption of groups for the reduction of metal ions and their oxidation to aldehydes, ketones, quinones, carboxylic acids, is associated with a concomitant decrease in hydrogen–bonded groups, leading to a shift towards higher wavenumbers. Consequently, additional bands of C=O groups may appear in the spectra of NPs in comparison to those of pure extracts, and/or the intensity of these bands may increase. Aldehyde C=O groups present, e.g., in reducing sugars may be oxidized to carboxyl groups by metal ions, resulting in the appearance of carboxyl group bands in the spectra of NPs [64]. In turn, the shift of C=O stretching may suggest that the biomolecules bind to NPs through this group, providing electrostatic stabilization of NPs. Analysis of the FTIR spectra allow us to demonstrate the involvement of diverse C=O groups on the surface of NPs, thereby preventing their aggregation and maintaining their stability during the aqueous phase. For instance, the splitting of a band of amide I into two bands indicate a change in the secondary structure of proteins, i.e., the involvement of these groups in the NPs’ formation (reduction and/or stabilization). The shift or disappearance of C–O–C, C–N, N–H and C–H bands also confirms the involvement of these functional groups in NPs’ formation and stabilization. In turn, the increase in amide II band intensity, may indicate the coverage of the NPs with those groups and their stabilization. Any changes in molecular geometry may lead to variations in vibrational frequencies due to differences in steric interactions and electronic environments surrounding the bonds. The molecule’s stiffening, a consequence of its stabilizing role in the NPs’ formation, may result in the disappearance of deformation vibrations or a shift of the stretching vibration bands of the C–H groups.

## 4. Challenges in the Interpretation of FTIR Spectra of Nanoparticles

There is a certain degree of subjectivity and interpretative flexibility inherent to the interpretation of FTIR spectra of NPs within the literature. A review of the literature reveals numerous examples where the same peak with a comparable wavenumber has been attributed to disparate functional groups, dependent on the author’s perspective. This phenomenon persists even when the chemical composition of the compounds under examination is analogous. While a comprehensive and precise interpretation of IR spectra of complex mixtures or complex compounds is unfeasible due to the presence of multiple functional groups in the IR spectra vibrating at similar wavenumbers, overly flexible interpretation can lead to confusion and errors in assigning peaks to individual functional groups, i.e., in the study of groups responsible for bio–reduction, capping and stabilization of nanoparticles. There are many publications where FTIR spectroscopy is employed for valid purposes, yet the spectra are misinterpreted. This primarily pertains to the assignment of bands to N–H and C–N groups, whose intensities in the spectra are typically of medium or low intensity [163]. The bands of N–H groups are visible in IR spectra within 3550–3250 cm^−1^ (aliphatic amines) and 3420–3340 cm^−1^ (aromatic amines) and in these ranges they may overlap with the bands of O–H stretching of –COOH groups in carboxylic acids (3300–2500 cm^−1^), O–H of alcohols (3670–3230 cm^−1^) and O–H in phenolic compounds (unassociated within 3620–3590 cm^−1^; associated within 3250–3000 cm^−1^). Medium to strong intensity vibrations of N–H groups vibration in 1° amines and amides in the 1650–1580 cm^−1^ range, medium–weak vibrations in 2° amines within 1580–1490 cm^−1^ and may be masked by aromatic ring C=C vibrations within 1625–1575 cm^−1^, while in the range 1295–1080 cm^−1^ (NH_2_ rocking/twisting in amines or amides) can be mistaken with C–O deformation and O–H stretching combination vibrations in phenols (1410–1310 cm^−1^; 1260–1180 cm^−1^) or C–H symmetrical deformation vibrations (1390–1340 cm^−1^). Similar situation occurs in the case of medium intensity C–N bond vibrations (1240–1020 cm^−1^, band position contingent upon the existence of neighbouring groups).

Assigning the vibrations of aromatic structures to specific bands in IR spectrum is also a challenge. Usually, the strongest absorptions for aromatic compounds occur within 3080–3010 cm^−1^ (stretching vibrations of the ring C–H bonds) and within 900–650 cm^−1^ (out of plane C–H vibrations of aromatic ring). Ring C=C stretching vibrations occur within 1625–1430 cm^−1^, but a weak band ~1000 cm^−1^ may also be observed, as well as weak combination and overtone bands within 2000–1650 cm^−1^. When an aromatic ring has a substituent, the bond between the carbon and the substituent is affected by the mass of this substituent and, consequently, a shift in the IR spectrum will be observed. In the case of phenolic compounds, the presence of additional bands originating from O–H and C–O bonds vibrations becomes evident. The position of these bands is also contingent upon the existence of substituents on the aromatic ring or the amount of phenolic moieties. Furthermore, if strong intramolecular hydrogen bonding occurs (e.g., in the presence of C=O groups), the band at ~1200 cm^−1^ may be found; however, this is dependent upon the presence and position of substituents in the aromatic ring (e.g., in ortho position). Consequently, the position of the O–H group band may change [163].

In light of the intricate composition of the extracts, the interpretation of their FTIR spectra represents a challenging endeavour, hindering the ability to draw definitive conclusions regarding the chemical nature and interactions occurring within the nanoparticles. Consequently, it is essential to approach the interpretation of the results with a meticulous attention. Overlapping peaks complicate the identification of specific functional groups in FTIR spectra and can lead to misinterpretation of the data. As it was mentioned, the use of second derivatives of the spectra can be a fruitful approach for the purpose of revealing unresolved bands [35,37]. Additionally, the implementation of machine learning algorithms to analyze FTIR spectra can assist in deconvoluting complex spectra and identifying overlapping peaks [39,40]. Nevertheless, it is always advisable to correlate the results obtained from the use of FTIR spectroscopy with those obtained by alternative techniques in order to ensure a comprehensive and informed understanding of the experimental findings.

## 5. Summary

The green synthesis of metal nanoparticles employs a variety of chemical functional groups that act as reducing, capping and stabilizing agents. Hydroxyl groups (–OH) can donate electrons to metal ions, reducing them to their elemental forms, and may act as both a reducing and stabilizing agent (hydrogen bonding). Carboxyl groups (–COOH) present in many organic acids and amino acids participate in the reduction process, enhance the solubility of nanoparticles in aqueous solutions and prevent agglomeration by providing steric hindrance. Amine groups (–NH_2_) facilitate electron transfer during the reduction of metal ions and this makes them effective reducing agents in nanoparticle synthesis also providing stability to the NPs surface. Aldehyde or ketone functionalities can effectively reduce metal ions and help in stabilizing formed NPs by creating a protective layer around them. Carbonyl groups (C=O) of ketones and aldehydes, present in various organic compounds and proteins, can facilitate complex formation with metal ions which is essential for controlling NPs size and morphology during synthesis. The analysis of these and a multitude of other functional groups can be effectively conducted through the utilization of FTIR spectroscopy.

This review presented information regarding application of FTIR spectroscopy in analysis of green synthesized nanoparticles and interpretation of infrared spectra with particular emphasis on the assignments of individual functional groups. Considering the potential of nanotechnology applications in everyday life and its dynamic development, it is necessary to assure detailed information about the chemical composition, functional groups involved in synthesis and molecular interactions in nanomaterials chemistry. FTIR spectroscopy offers a suite of advantages for nanoparticle analysis, including non–destructive testing, detailed surface characterization, high sensitivity and resolution, versatility across materials and the ability to monitor synthesis processes. These features make it an invaluable tool in nanotechnology research and development.

## Figures and Tables

**Table 1:** Advantages and limitations of TS, ATR and PAS techniques.

FTIR Technique	Advantages	Limitations
TS	provides high quality spectra with good resolution high S/N ratio does not require any additional accessories signal is derived from the entire bulk of the sample	requires significant sample preparation (grinding, diluting with KBr, pellets) risk of samples contamination during grinding with KBr results can vary based on pellet thickness and uniformity no possibility of sample recovery not suitable for optically opaque samples
ATR	minimal sample preparation required non–destructive analysis quick and flexible; suitable for a wide range of sample types including solids and liquids IR signal penetrates to a depth of μm within the sample	good contact between the sample and the ATR crystal is required worse S/N ratio than TS provides information mainly from the surface of the sample possible relative shift in the intensity of the bands and in vibrational frequencies
PAS	minimal sample preparation required useful for opaque or highly absorbing samples IR signal penetrates to a depth of μm within the sample	relatively poor S/N ratio (longer data acquisition required) tested samples must be dry

**Table 2:** Spectral positions of infrared absorption bands of metal oxides.

Metal Oxide	1400–700 cm^−1^	600 cm^−1^	500 cm^−1^	400 cm^−1^	Ref.
CaO	1081 *, 714	–	–	400	[139,140]
CoO	–	662, 626	555	–	[139,141,142]
Co_3_O_4_	–	670	585, 558	–	[139,141]
CuO	–	660, 600	540	490, 430	[139,143]
FeO	1125	–	–	410	[139,144]
α–Fe_2_O_3_	1110, 1030	–	543	472	[139,145]
Fe_3_O_4_	–	620	580	–	[139,146,147]
NiO	–	–	–	448, 410	[139,148]
SnO	–	690, 620	–	–	[139]
SnO_2_	1400, 1385	620, 600	–	–	[139,149]
TiO_2_					
anatase	800	685, 650, 602	551	431	[139,150,151,152,153]
rutile		678	500	449	[153]
ZnO	878	618	554, 530	498, 440	[139,154,155,156]

* band of carbonate anion.

**Table 3:** Wavenumber ranges of vibrations of functional groups, involved in reduction, capping and stabilization of nanoparticles [64,74,94,95,98,99,100,101,102,103,104,105,106,107,108,109,110,111,112,113,114,115,116,117,118,119,120,121,122,127,128,129,130,131,132,133,134,135,140,142,143,144,145,146,147,148,149,150,151,152,153,154,155,156,157,158,159,164,166,173,174,175,176,177,178,179,182,185,186].

Wavenumber (cm^−1^)	Band Assignment
3730–3520	free –OH str.
3600–3100	OH of water (broad)
3550–3230	hydrogen–bonded –OH str. (broad)
3550–3330	NH_2_ asym. str. (1° amines)
3450–3160	NH_2_ sym. str. (1° amines)
3540–3480 3420–3380	1° amides (–CO–NH_2_); N–H asym. str.
3370–3270	2° amides, N–H str.
3370–3270	O–H, N–H (1° amines, amides)
3105–3000	aromatic =C–H and ring C=C vib.
3095–3075	=CH_2_ (alkene)
2975–2950	–CH_3_ asym. str.
2885–2865	–CH_3_ sym. str.
2940–2915	–CH_2_– asym. str.
2870–2840	–CH_2_– sym. str.
2260–2200	–C≡N (nitriles)
2260–2240	–OCN str. (cyanates)
2300–2250	–N=C=O asym. str. (isocyanates)
1850–1740	C=O (carboxylic acid anhydride)
1740–1650	C=O (carboxylic acids)
1745–1715	C=O (aliphatic aldehydes, ketones, esters)
1700–1680	C=O (aromatic aldehydes, ketones, esters)
1700–1600	C=O str., amide I * band
1695–1540	COO^−^ asym. str. (carboxylic acid salts)
1690–1605	C=O (quinones)
~1660	aromatic ring C=C str. (phenol)
1650–1580	N–H def. vib. (1° amines, aromatic amines, amides)
1630–1600	OH of water
1625–1525	aromatic =C–H and ring C=C vib.
1620–1610	C=C stretching (alkene)
1580–1490	N–H def. vib. (2° amines)
1570–1510	N–H b. and C–N str. in –CO–NH– (amide II **)
1465–1430	–CH_3_ asym. b.
1465–1430	aromatic C=C stretching
1450–1440	N–H def. vib. (amides)
1440–1260	in–plane O–H def. vib. (alcohol)
1440–1335	COO^−^ sym. str. (carboxylic acid salts)
1440–1395	combination of O–H def. and C–O str. (carboxylic acids)
1420–1400	C–N str. (amide III ***) (1° amides)
1410–1310 1260–1180	combination of O–H def. and C–O str. (phenol)
1390–1370	–CH_3_ sym. b. (characteristic of C–CH_3_)
1380–1280	O–H def. vib. (carboxylic acids)
1360–1250	C–N str. (aromatic amines)
1310–1250	C–O–C asym. str. (esters)
1305–1200 1190–1170 1145–1130	C–N str. (amide III ***) (2° amines)
1190–1075	C–O str. (carboxylic acids)
1175–1165	C–C skeletal str. (alkanes)
~1110	aromatic C–H def. vib. in phenolic compounds
1150–1060	C–O–C str. (ethers, esters)
1090–1000	CCO str. (alcohol)
1090–1020	C–N str. (1° amines)
1075–1000	CO str. (alcohol)
960–875	out–of–plane O–H def. vib. (carboxylic acids)
960–800	CH_2_ twisting vib. (alcohol)
895–650	N–H out–of–plane b. vib. (1° amines, aromatic amines, amides)
820–770	combination of O–H def. and C–O str. (phenol)
820–670	aromatic =C–H and ring out–of–plane vib.
785–720	–CH_2_– rocking vib.
765–690	C–H out–of–plane def. vib., aromatic ring def. vib.
750–700	N–H wagging vib. (2° amines), broad
750–600	NH_2_ def. vib. (1° amides), broad
720–600	out–of–plane O–H def. vib. (phenol)
710–570	out–of–plane O–H def. vib. (alcohol)
630–580	in–plane C–CO–C def. vib. (aliphatic aldehydes, ketones, aromatic methyl aldehydes, ketones)
600–550	N–C=O def. vib. (1° amides)
500–450	C–C=O def. vib. (1° amides)

str.—stretching, b.—bending, def.—deformation, vib.—vibrations, * amide I—major contribution of C=O vib., ** amide II—N–H def. and C–N str. vib., *** amide III—C–N vib.

## Data Availability

Not applicable.

## Abbreviations

AFM	Atomic force microscopy
ATR	Attenuated total reflectance
DLS	Dynamic light scattering
EDX	Energy–dispersive X–ray spectroscopy
FIR	Far–infrared spectroscopy
FTIR	Fourier Transform Infrared spectroscopy
IR	Infrared
HRI	High refractive index
NMR	Nuclear magnetic resonance
NIR	Near–infrared spectroscopy
NPs	Nanoparticles
PAS	Photoacoustic spectroscopy
PCA	Principal Component Analysis
PLS	Partial Least Squares
ROS	reactive oxygen species
SEM	Scanning electron microscopy
S/N	signal–to–noise ratio
TEM	Transmission electron microscopy
UV–Vis	Ultraviolet–visible spectrophotometry
XPS	X–ray photoelectron spectroscopy
XRD	X–ray diffraction
